# Effects of kangaroo mother care on feeding intolerance in preterm infants

**DOI:** 10.1093/tropej/fmad015

**Published:** 2023-03-10

**Authors:** Sinem Yalnızoğlu Çaka, Sümeyra Topal, Sadık Yurttutan, Selin Aytemiz, Yasemin Çıkar, Murat Sarı

**Affiliations:** Department of Pediatric Nursing, Faculty of Health Sciences, Kocaeli University, Kocaeli 41001, Turkey; Department of Pediatric Nursing, Faculty of Health Sciences, Kahramanmaraş İstiklal University, Kahramanmaraş 46100, Turkey; Division of Neonatology, Department of Pediatrics, Faculty of Medicine, Kahramanmaras Sutcu Imam University, Kahramanmaraş 46040, Turkey; Department of Neonatal Intensive Care Unit, Kahramanmaras Sutcu Imam University Health Practice and Research Hospital, Kahramanmaraş 46040, Turkey; Department of Neonatal Intensive Care Unit, Kahramanmaras Sutcu Imam University Health Practice and Research Hospital, Kahramanmaraş 46040, Turkey; Department of Neonatal Intensive Care Unit, Kahramanmaras Sutcu Imam University Health Practice and Research Hospital, Kahramanmaraş 46040, Turkey

**Keywords:** preterm, kangaroo mother care, preterm feeding intolerance, gastric residual, nursing care

## Abstract

**Objective:**

Feeding intolerance (FI) is a common condition in preterm infants because they have an immature gastrointestinal tract. There are studies on the effects of the position on gastric residual volume (GRV) in preterm infants. Kangaroo mother care (KMC) may be an instrument for reducing FI by providing an upright position to infants. Moreover, numerous studies conducted with this therapeutic position applied by putting an infant on the mother’s chest have indicated its positive effects on the infant’s weight gain, growth and development, and vital signs. Therefore, this study aimed to reveal the impact of KMC on FI in preterm infants.

**Methods:**

The population of the study, designed as a randomized trial, consisted of 168 preterm infants [KMC: 84, Standart Care (SC): 84] hospitalized in the neonatal intensive care unit of a university hospital between June and November 2020. Infants were randomly selected and divided into two groups. After the vital signs of the infants in both groups became stable, the infants were fed in the same position. KMC was applied to the infants in the intervention group for 1 h by preparing a suitable environment after feeding. Infants in the SC group were placed in the prone position after feeding. The GRVs of the infants in both groups were recorded on the Infant Follow-up Form before the next feeding.

**Results:**

No statistically significant difference was detected between the groups upon comparing them in terms of demographic and clinical characteristics. The body temperatures and O_2_ saturations of the participants in the KMC group were statistically significantly higher, and their respiratory and heart rates were lower than the SC group. The transition time to full enteral feeding was statistically significantly shorter, and FI was experienced significantly less in the KMC group infants than in the SC group (*p* < 0.05). There was no statistically significant difference between the groups in terms of the infants' weight gain and length of hospital stay (*p* > 0.05).

**Conclusion:**

The present study demonstrated that KMC had a positive impact on FI in preterm infants. KMC is not only a safe care model providing the earliest contact between parents and infants but also a practice whose positive effect on the functioning of the digestive system in preterm infants we can use.

## INTRODUCTION

Enteral feeding plays an essential part in the survival of preterm infants [1]. Infants born before 34 weeks of gestation are at risk of aspiration due to the lack of coordination between sucking, swallowing, and breathing. Hence, these infants should primarily be fed with gavage (enteral) feeding. Providing nutritional support equivalent to the growth and development rate of the intrauterine period is one of the care team's goals during this period [2]. Feeding intolerance (FI) is a severe problem with significant adverse impacts in preterm infants [3, 4]. FI represents a common phenomenon in neonatal intensive care units and occurs in 16–29% of preterm infants [5, 6]. FI is defined as the disruption of the patient’s feeding plan and the inability to digest enteral food, manifested as a gastric residual volume (GRV) higher than 50%, abdominal distention/vomiting or both [7]. Studies emphasize that clinical/abdominal examination findings, stomach contents, laboratory and radiological findings are indicative for the detection of FI [7–9]. Abdominal examination findings (distention, an increased abdominal circumference, prominent bowel loops, an increase in/absence of bowel sounds, vomiting, >50% and/or bloody residues if checked) and changes in defecation frequency without a significant deterioration in clinical condition (such as apnea and hypotension) can be observed as clinical findings [8, 9]. In preterm infants, FI may develop due to decreased GI motility and even potentially progress to necrotizing enterocolitis (NEC), a cause of premature death [3].

Feeding-related nursing care has been accepted as a key factor in increasing the survival rates, health and development of infants with special care needs [10]. Deciding on which position after feeding will lead to more positive outcomes for an infant also causes confusion among caregivers. Although there are numerous studies reporting that the body position impacts gastric emptying or residual volume in preterm infants [11–16], a consensus on the appropriate position that should be given to the preterm infant after feeding to reduce GRV has not been reached yet. Additionally, several studies report that holding the infant in the prone or right lateral position after feeding reduces GRV and regurgitation [14–16].

Kangaroo mother care (KMC) is a care approach within the scope of developmental care theory, which is used the most to support these infants, and it has many physiological and behavioral benefits [17, 18]. A significant advantage of KMC is its potential to reduce FI by providing infants with a prone and upright position. KMC is a tool in this regard, but it does not only have this advantage. Many studies conducted with this therapeutic position, applied by putting an infant on the mother's chest, have demonstrated its positive effects on the infant’s weight gain, growth and development, and vital signs [19–21]. Since no study was found on the effects of KMC on feeding tolerance in preterm infants, the current research was conducted to reveal the impacts of KMC on feeding tolerance in preterm infants. Based on the above literature review, our hypothesis is: Preterm infants to whom KMC (skin-to-skin contact) is applied after feeding experience less FI than preterm infants to whom routine feeding is applied.

## METHODS

### Study design

The research was designed as an experimental trial to demonstrate the effects of KMC on FI in preterm infants. The study population comprised all preterm infants (*N*: 289) hospitalized in the neonatal care unit of a hospital in Kahramanmaraş between June and November 2020, and the sample comprised 168 preterm infants [KMC: 84, Standart Care (SC):84] who met the inclusion criteria.

#### Sample selection criteria

Preterm infants who were born between 28 and 36 gestational weeks determined according to the time of mothers' last menstruation and obstetric evaluation results, had low birth weight (weighed between 1000 and 2500 g) during the research, had stable vital signs and could consume 75% of the total protein and energy through an orogastric (OG) tube, were fed with breast milk and eoprotein, did not use muscle relaxants, analgesics, sedatives or inotropic drugs, did not have a severe neurological disease and breathed spontaneously were included in the study. KMC was applied by non-smoking mothers volunteering to take part in the research.

#### Exclusion criteria

Intubated preterm infants without cardiorespiratory stabilization and with pneumothorax, vomiting or bilious-stained gastric aspirate, other gastrointestinal diseases, NEC, skull fracture, severe atelectasis or a history of surgery that might impact their comfort, and a condition that prevented the placement of a chest tube or parent's/infant's KMC position were not included in the research. Furthermore, mothers not speaking Turkish were not enrolled in the study.

**Figure 1:**
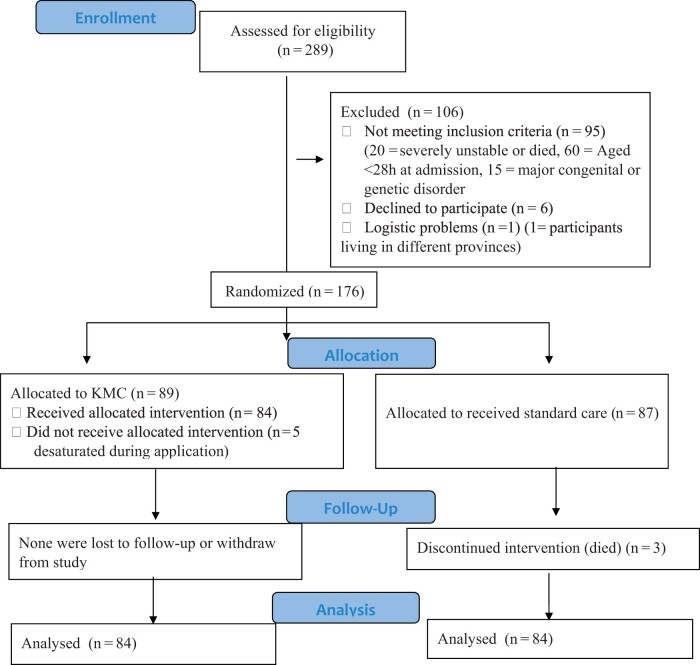
Flow chart of research.

Power analysis was performed in the G*Power (3.1.9.2) program for the purpose of finding the sample number. The effect size (d) of 0.555 was computed using the mean (0.9 and 2.0) and standard deviations (1.6 and 2.3) obtained from the relevant literature [9]. According to the formula specified above, the required minimum sample size was 140 preterm infants, 70 for each group. The study was completed with 168 preterm infants (KMC: 84, SC: 84) by taking into account possible case losses (Figure 1).

The urn method, which represents a method of randomization that is equivalent to the full randomization method, was employed to ensure the randomization of the groups. Two parameters, *α* and *β*, are mentioned in this method. The said parameters represent red and white balls. There is a possibility of A to be white or red. B represents balls of the opposite color to the ball we selected in *α*. One of the balls is selected in a random way, and if the color of the chosen ball is white, the person is assigned to group A, and if its color is red, the person is assigned to group B. The above-mentioned process is repeated during each assignment [22].

### Data collection

To collect the study data, the Descriptive Information Form, KMC Follow-up Form, and GRV Measurement Form were used.

#### Descriptive information form

The form in question contains the descriptive information (age, weight, sex, etc.) of the mother and the infant.

#### KMC follow-up form

Information such as the date of starting KMC, vital signs measured before and after KMC, and the amount of residue (ml) measured before the next feeding (approximately in a 3-h period) is recorded.

#### GRV measurement form

‘FI’ represents a combination of clinical signs that suggest that an individual cannot tolerate enteral feeding. The most comprehensive definition is presented below: It is the inability to digest more than 50% of enteral feeding presented as GRV, abdominal distention or vomiting, or both of them, and thus disruption of the patient’s feeding plan [7]. The feeding of preterm infants hospitalized in the unit and their FI are evaluated according to the Turkish Neonatology Association Nutrition Guide for Preterm and Sick Term Infants (2018) [3]. According to this guide, the presence of >50% and/or bloody residue, if checked, is among the FI findings.

An OG or nasogastric (NG) tube suitable for the week of gestation was inserted at the time of hospitalization. In the current research, preterm infants were fed by the intermittent feeding method every 3 h; the feeding time was 30 min with a pump. GRV was checked in infants with abdominal distention or vomiting before the previous feeding (measuring the gastric content after feeding and resting using an injector), and residuals >50% and/or bloody residues were considered FI.

#### Procedure

- Before including them in the study, the researcher met with the family and the infant. The study's aim and KMC application were explained to the family. An informed consent form was given to the families who wanted to take part in the study, and the family read it and approved it. The infant's group was determined according to the urn method. The Mother and Infant Descriptive Information Form among the data collection forms was filled out.
- All interventions in the KMC and SC groups were performed by two independent observers simultaneously, YÇ (also a nutrition nurse) and MS (also an infant care nurse), who are among the study authors and are currently working in the day shift in the Neonatal Intensive Care Unit.
- Enteral (with OGT/NGT) nutrition is started in preterm infants with a gestational age of <3234 weeks, with sucking/swallowing dysfunction, or who cannot be fed orally. If the infant's weight is between 1000 and 1500 g, it is increased to feed the infant as 15–20 ml/kg/day (for 1–2 days) and then 30 ml/kg/day every 2–3 h. If the infant's weight is between 1500 and 1800 g, he/she is fed as 20 ml/kg for 1 day and then 30 ml/kg/day every 3 h. Breast milk fortification is initiated when feeding reaches 50–100 ml/kg (recommended 80 ml/kg) [3].
- The breast milk of mothers with premature babies may be insufficient in the first days. Therefore, as breast milk increases, the formula is stopped and the mother's expressed breast milk is enriched with eoprotein. In order to evaluate the effect on the infant's feeding tolerance, KMC was performed when the mother's milk reached a sufficient level for her baby (15–20 cc breast milk in each expressing) (72 h after the earliest birth). Hence, KMC was carried out while infants were fed only with breast milk. Minimal enteral nutrition is started with colostrum. Breast milk is expressed at least 6 times, preferably 8–12 times, and given to the infant with an OG/NG tube.
- Enteral feeding is stopped in case of severe respiratory distress (respiratory rate>80/min), GIS obstructions due to congenital malformations, diagnosis of NEC, hemodynamics, irregularity and shock requiring high inotropic treatment support, or multiple organ failure.
- Infants in both groups were fed in the supine position with their heads raised at 30–45° after the position control in preterm infants with an OG or NG tube inserted.
- Before the procedure, eoprotein was added to the breast milk of the preterm infant (one measure of eoprotein to 25 cc of breast milk) and heated in a standard food warmer. Feed volumes were the same for each infant.
- The vital signs of the infants in both groups were checked before feeding, and the study was continued with stable (heart rate 100–160 beats/min, respiration rate <60/min, and axillary temperature 36.5–37.4°C) infants [23]. Feeding was performed with a perfusor in 30 min.
- KMC was applied to the infants in the intervention group with stable vital signs after feeding for 60 min [Overall mean time for initiate KMC is 3.74 ± 0.79 days (min: 3, max: 5)]. KMC is the practice of placing an infant with only a hat and diaper on the parent's open chest in a prone, upright position (approximately 45°) and providing skin-to-skin contact.
- KMC was applied on one of the visiting days at 14.00. Attention was paid to the mother's privacy.
- In the hospital routine, a nest is created with rolled up cloths in the incubator (the newborn's hand-foot coordination is supported by the nest, and stress and energy consumption are reduced due to a decrease in swaying and involuntary movements).
- To ensure homogeneity between the groups and evaluate the effectiveness of KMC, while infants in the intervention group received KMC after feeding, infants in the SC group were placed in the prone position after feeding. The prone position after feeding is currently practiced in infants receiving standard care in the ward. When studies assessing the effect of the position given after feeding on GRV in newborns are reviewed, it has been proven that the prone position is superior to other positions with low GRV and high nutrient absorption probability [11–14]. On the other hand, infants lying in a prone position are at risk of Sudden Infant Death Syndrome. Therefore, respiration and O_2_ saturation of infants in the prone position were carefully monitored. At the same time, to prove that the difference between our hypothesized groups was skin-to-skin contact, infants in the SC group were placed in the prone position at an angle of approximately 45° and in the rolled nest by placing an inclined reflux bed inside the incubator as if they were lying on the mother's lap.
- Infants in the standard care group were placed in the prone position after feeding.
- YÇ and MS checked vital signs after 60 min in both groups. They evaluated the residual volume of infants in both groups prior to the next feeding (approximately 3 h later).
- All interventions were performed under the same environmental conditions (quiet, calm and low-lit environment).

### Ethical considerations

The study was approved by the Clinical Studies Ethics Committee of Kahramanmaraş Sütçü İmam University in Turkey (Date: 2020/24, Decision No: 03). Written informed consent was obtained from all participants before enrollment into the study.

## STATISTICS

In the research, the data of 168 participants were assessed and transferred to IBM SPSS Statistics 23 program on the computer. The participants' descriptive characteristics were analyzed with frequency (*n*, %) for categorical variables and mean and standard deviation for continuous variables. In the evaluation of the study data, the Kolmogorov–Smirnov test was conducted to reveal the status of the normal distribution in the groups [24]. Pearson's chi-square test was performed to compare qualitative data. The independent sample t-test was conducted to investigate the difference between two-group discontinuous variables. Statistical significance was regarded to be *p* < 0.05.

## RESULTS

When the participants in the KMC and SC groups were compared in terms of some descriptive and clinical characteristics, the groups did not differ statistically significantly (*p* > 0.05) (Table 1).

**Table 1.** Comparison of some descriptive and clinical characteristics by groups

		KMC group (*n* = 84)	SC group (*n* = 84)	Test value	*p*
		Mean ± SD	Mean ± SD		
Week of gestation (weeks)		32.10 ± 2.82	32.78 ± 2.51	*t* = −1.560	0.121
Gravida		2.69 ± 1.74	2.60 ± 1.48	*t* = 0.334	0.739
Parity		2.45 ± 1.49	2.38 ± 1.15	*t* = 0.347	0.729
Birth weight (g)		1756 ± 503	1901 ± 557	*t* = −1.764	0.079
Birth length (cm)		42.70 ± 3.61	43.54 ± 2.95	*t* = −1.661	0.099
Head circumference at birth (cm)		30.18 ± 2.40	30.60 ± 2.15	*t* = −1.190	0.236
First minute APGAR score		6.5 ± 1.3	6.8 ± 1.	*t* = −1.331	0.185
Fifth minute APGAR score		8.1 ± 1.30	8.3±.95	*t* = −1.219	0.225

		***n* (%)**	***n* (%)**		

Sex	Female	43 (49.4)	44 (50.6)	*χ* ^2^ = 0.024	0.877
	Male	41 (50.6)	40 (49.4)		
Birth mode	Cesarean section	77 (49.4)	79 (50.6)	*χ* ^2^ = 0.359	0.549
	Normal	7 (58.3)	5 (41.7)		
Small for gestational age (SGA)	Yes	35 (56.6)	28 (44.4)	*χ* ^2^ = 1.227	0.268
	No	51 (46.8)	58 (53.2)		
History of infection during pregnancy	Yes	11 (45.8)	13 (54.2)	*χ* ^2^ = 0.194	0.659
	No	73 (50.7)	71 (49.3)		
History of EMR	Yes	9 (39.1)	14 (60.9)	*χ* ^2^ = 1.259	0.262
	No	75 (51.7)	70 (48.3)		
Use of a mechanical ventilator	Yes	28 (42.4)	38 (57.6)	*χ* ^2^ = 2.469	0.114
	No	56 (54.9)	46 (45.1)		
Use of surfactant	Yes	19 (54.3)	16 (45.7)	*χ* ^2^ = 0.325	0.569
	No	65 (48.9)	68 (51.1)		
Presence of sepsis	Yes	8 (34.8)	15 (65.2)	*χ* ^2^ = 2.468	0.116
	No	76 (52.4)	69 (47.6)		

*t*, Student’s *t*-test; *χ*^2^, Pearson’s chi-square test; EMR, early membrane rupture.

* *p* < 0.05.

** *p* < 0.001.

When the participants in the groups were compared in terms of pre-feeding body temperatures, respiratory rates, O_2_ saturations and heart rates, no statistically significant difference was determined between them. When body temperatures, respiratory rates, O_2_ saturations and heart rates of the newborns in the KMC and SC groups at the 60th minute after feeding were compared, a statistically significant difference was found between them (*p* < 0.001). Accordingly, it was determined that the body temperatures and O_2_ saturations of the participants in the KMC group were statistically significantly higher, and their respiratory and heart rates were lower than the SC group. When the participants in the KMC and SC groups were compared according to weight gain, feeding and length of hospital stay, it was determined that the transition time to full enteral feeding was statistically significantly shorter in the KMC group infants than those in the SC group (*p* < 0.05). There was no statistically significant difference between the groups in terms of the infants' weight gain and length of hospital stay (*p* > 0.05) (Table 2).

**Table 2.** Comparison of vital signs, weight gain, feeding and length of hospital stay in preterm newborns by groups

Variables	KMC group (*n* = 84)	SC group (*n* = 84)	*t*-test	*p*
	Mean ± SD	Mean ± SD		
Body temperature (°C)				
Before feeding	36.59 ± .26	36.55 ± −0.23	1.075	*0.284*
60 min after feeding	36.66 ± .18	36.56 ± 0.18	3.748	0.000**
Test value^a^/*p*	*t*: −3.206/0.002**	*t* = −0.370/*0.712*		
Respiratory rates (min)				
Before feeding	50.47 ± 3.88	50.32 ± 3.39	0.272	*0.786*
60 min after feeding	45.50 ± 3.22	50.13 ± 3.51	−9.016	0.000**
Test value^a^/*p*	t: 28.561/0.000**	t: 1.111/*0.270*		
O_2_ saturation (%)				
Before feeding	94.87 ± 1.17	95.10 ± 1.20	−1.279	*0.203*
60 min after feeding	97.76 ± 0.83	95.06 ± 0.64	23.673	0.000**
Test value^a^/*p*	*t* = −30.458/0.000**	*t* = 0.259/*0.796*		
Heart rate (min)				
Before feeding	137.76 ± 6.92	135.98 ± 7.21	1.650	*0.101*
60 min after feeding	132.05 ± 5.67	136.02 ± 7.25	−3.994	0.000**
Test value^a^/p	*t* = 23.385/0.000**	*t* = −0.251/*0.802*		
Average weight gain per day (g)	7.67 ± 11.72	4.84 ± 15.33	1.361	*0.175*
Weight on the 10th day (g)	1785.77 ± 516.91	1881.01 ± 537.82	−1.170	*0.244*
Weight at discharge (g)	2082.73 ± 361.03	2189.46 ± 368.60	−1.896	*0.060*
Time of transition to full enteral feeding (days)	7.82 ± 5.30	10.04 ± 8.21	−2.086	0.039*
Length of hospital stay (days)	26.77 ± 20.14	24.51 ± 18.11	.765	*0.445*

*t*, Student’s *t*-test.

a Paired sample *t*-test.

^*^ P-values indicate significance <0.05 are represented in Bold.

^**^ P-values indicate significance < 0.001 are represented in Bold.

Upon comparing the participants in the KMC and SC groups according to the presence of FI, a statistically significant difference was revealed between the groups (*p* < 0.05). When the GRV of the participants was compared, it was determined that the amount of food they took just before the intervention was 35.44 ± 8.42 in the KMC group and 36.70 ± 8.62 in the SC group, and the difference was statistically insignificant (*p* > 0.05). In the preterm infants, the mean residual volume measured before the next feeding was 0.84 ± 3.28 in the KMC group and 4.17 ± 6.04 ml in the SC group. Accordingly, the participants in the KMC group were significantly less likely to experience FI than those in the SC group (*p* < 0.05) (Table 3).

**Table 3.** Comparison of feeding intolerance in preterm newborns by groups

Feeding intolerance		KMC group (*n *= 84)	SC group (*n* = 84)	*χ* ^2^ test value	*p*
		*n* (%)	*n* (%)		
Abdominal distention or vomiting + >% 50 and/or blood residues	Yes	4 (18.2)	18 (81.8)	10.252	0.001**
	No	80 (54.8)	66 (45.2)		

		**Mean ± SD**	**Mean ± SD**	***t*-test**	***p***

Enteral breast feeding volume (ml)		35.44 ± 8.42	36.70 ± 8.62	−0.975	*0.331*
GRV before next feeding (ml)		0.84 ± 3.28	4.17 ± 6.04	−4.484	0.000**

*χ* ^2^, Pearson’s chi-square test; *t*, Student’s *t*-test; GRV, gastric residual volume.

^*^ P-values indicate significance <0.05 are represented in Bold.

^**^ P-values indicate significance < 0.001 are represented in Bold.

## DISCUSSION

FI represents a group of clinical symptoms of abnormal feeding and disorders caused by gastrointestinal dysfunction due to different disease factors in the neonatal period [25]. It is stated that the progression of FI to NEC can be prevented by comprehensive nursing evaluations and early interventions [26].

Nutrition care is one of the primary measures to save preterm infants. In this regard, proper positioning is one of the key measures that is done by nurses; still there is a paucity of studies on the impacts of different body positions applied to preterm and low birth weight infants on residual gavage volume and gastric emptying time and the results of these few studies are an area of ongoing debates [11, 14, 16]. Some studies in the literature demonstrate that the KMC position applied in neonatal clinics during gavage feeding increases breast milk production and the infant's breastfeeding amount. In their study, Valizadeh, *et al.* [9] demonstrated that infants in the KMC position tolerated feeding better than in the supine position. The findings of the research by Törnhage, *et al.* [10] demonstrated that the plasma cholecystokinin level increased significantly and the plasma somatostatin level did not change in infants who were fed through a NG tube and to whom KMC was applied. Cholecystokinin is one of the important peptides of the digestive system, which is under the control of parasympathetic nerves and regulates the function of the digestive system by preventing gastrin secretion and increasing secretin secretion and stimulates peristatic movements [27]. Considering the present research findings, infants to whom KMC was applied experienced FI significantly less than infants in the SC group.

When the infants were compared their vital signs, weight gain, feeding, and length of hospital stay, it was determined that the transition time to full enteral feeding was statistically significantly shorter in the infants in the KMC group in comparison with the SC group. Additionally, the participants in the KMC group had higher body temperatures and O_2_ saturation and lower respiratory and heart rates than the SC group. When the weight gains and length of hospital stay were examined between the groups, it was found that the preterm infants in the intervention group had higher weight gain and shorter hospital stay than those in the SC group, but the difference was statistically insignificant. In this study, it can be said that since the infants to whom KMC was applied were placed in the middle of the breast and were in contact with the smell of breast milk, feeling themselves in a safe environment in the mother's womb positively affected their vital signs. Moreover, they tolerated feeding better, and the transition time from gavage feeding to oral feeding was shorter than other methods. Although the feeding of preterm infants has been studied for long years, there is still uncertainty about selecting and providing the most appropriate way to feed these infants. The research by Yıldız, *et al.* [28] also indicated that the transition time from gavage feeding to oral feeding was shorter and the average length of hospital stay was shorter in preterm infants stimulated by the smell of breast milk. Studies on this subject emphasize that kangaroo care shortens the length of hospital stay of preterm infants and has a direct positive effect on growth development, supporting our study [19, 20]. Therefore, when mothers participate in the feeding of preterm infants, not only infants benefit from this care, but it can also effectively reduce mothers' anxiety and contribute positively to the acceleration of discharge [29].

There were also some limitations of this study. First, FI in preterm infants is affected by multiple risk factors, including race, history of abnormal Doppler’s in pregnancy, some environmental factors that may alter the infant's bacterial flora/antibiotic use and the infant's diet. This study neglected the influence of these confounding factors. In addition, although only those who breastfed were included, the variability in the content of breast milk and the formula they used in the first days may also have affected the FI. Finally, this study has been performed in only one center.

In conclusion, KMC affected FI in preterm infants. During feeding, infants can be placed in the KMC position so that they benefit from maternal care while benefiting from the positive effect of this upright position on the digestive system’s functioning. In line with the findings and limitations of the current research, it is recommended to compare the effects of KMC with different positions in preterm infants in the NICU.

## FUNDING

None.
